# Prevalence and risk factors of sepsis-induced cardiomyopathy: A retrospective cohort study

**DOI:** 10.1097/MD.0000000000005031

**Published:** 2016-09-30

**Authors:** Ryota Sato, Akira Kuriyama, Tadaaki Takada, Michitaka Nasu, Sarah Kyuragi Luthe

**Affiliations:** a Department of Emergency and Critical Care Medicine, Urasoe General Hospital; b Department of General Medicine, Kurashiki Central Hospital; c Department of Anesthesia, Urasoe General Hospital, Okinawa, Japan.

**Keywords:** cardiac depression in sepsis, sepsis, sepsis-induced cardiomyopathy, septic cardiomyopathy, septic shock

## Abstract

The aim of the study is to evaluate the epidemiology and clinical features of sepsis-induced cardiomyopathy (SICM).

A retrospective cohort study was conducted.

A total of 210 adult patients with sepsis or septic shock admitted to a Japanese tertiary care hospital from January 1, 2013, to December 31, 2015, who underwent transthoracic echocardiography (TTE) on admission.

The definition of SICM was ejection fraction (EF) < 50% and a ≥10% decrease compared to the baseline EF which recovered within 2 weeks, in sepsis or septic shock patients.

Our primary outcome was the incidence rate of SICM. Our secondary outcomes were the in-hospital mortality rate and length of intensive care unit (ICU) stay according to the presence or absence of SICM. In total, 29 patients (13.8%) were diagnosed with SICM. The prevalence rate of SICM was significantly higher in male than in female (*P* = 0.02). Multivariate logistic regression analyses revealed that the incidence of SICM was associated with younger age (odds ratio [OR], 0.97; 95% confidence interval [CI], 0.95–0.99), higher lactate level on admission (OR, 1.18; 95% CI, 1.05–1.32) and history of heart failure (HF) (OR, 3.77; 95% CI, 1.37–10.40). There were no significant differences in the in-hospital and 30-day mortality between patients with and without SICM (24.1% vs 12.7%, *P* = 0.15; 20.7% vs 12.1%, *P* = 0.23). Lengths of hospital and ICU stay were significantly longer in patients with SICM than in those without SICM (median, 43 vs 26 days, *P* = 0.04; 9 vs 5 days, *P* < 0.01).

SICM developed in 13.8% of patients with sepsis and septic shock. A younger age, higher lactate levels on admission and history of HF were risk factors.

## 1 Introduction

Sepsis-induced cardiomyopathy (SICM) is a reversible myocardial depression due to sepsis and septic shock which was first reported by Parker et al^[1]^ in 1984. Recent developments in transthoracic echocardiography (TTE) have enabled the visualization of hemodynamics in SICM, characterized by left ventricular dilation, depressed ejection fraction (EF), and recovery in 7 to 10 days.^[2]^

Several studies have reported the pathogenesis of SICM. Chemical mediators including endotoxins, cytokines, and nitric oxide are considered to be the main mediators.^[3–5]^ However, the understanding remains incomplete. Furthermore, while SICM is relatively common, its epidemiology and clinical features remain unclear as well. Therefore, this study aimed to reveal the epidemiology and clinical features of SICM.

## 2 Method

We conducted a retrospective cohort study comprising adult patients with sepsis or septic shock, who were admitted to Urasoe General Hospital, a 311-bed Japanese tertiary care hospital, from January 1, 2013, to December 31, 2015. Patients who were performed TTE on admission were enrolled. In the ICU, sepsis was defined as patients with an acute change in the total Sequential Organ Failure Assessment (SOFA) score ≥ 2 points consequent to the infection. In out-of-hospital, emergency department (ED) and general hospital ward settings, quick SOFA (qSOFA) was used to identify sepsis. The qSOFA was defined as patients with infection who have at least 2 of the following criteria: respiratory rate of 22/min or greater, altered mentation, systolic blood pressure of 100 mm Hg or less. Septic shock was defined as vasopressor requirement to maintain a mean arterial blood pressure of 65 mm Hg or greater, and serum lactate level greater than 2 mmol/L in the absence of hypovolemia.^[6]^

**Figure 1:**
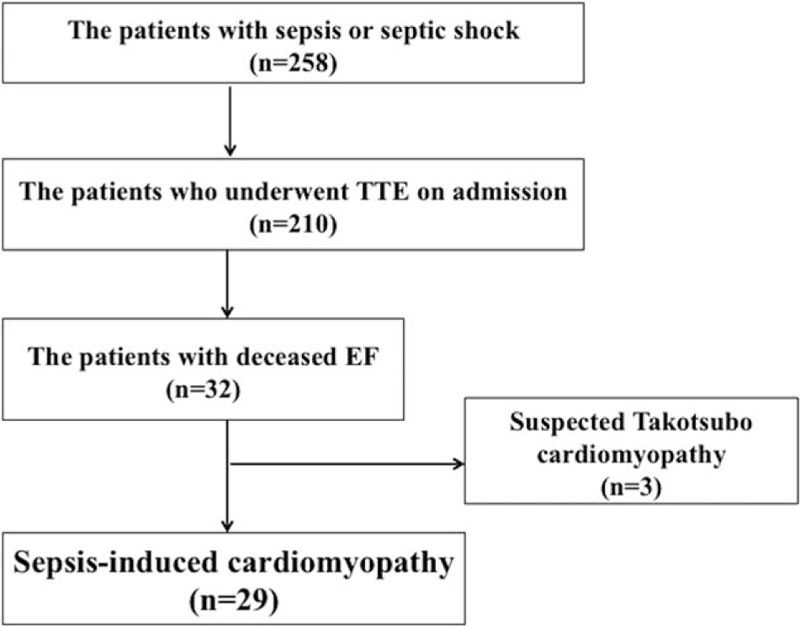
In total, 258 patients had sepsis or septic shock. Of these, 210 patients with sepsis or septic shock who underwent transthoracic echocardiography (TTE) on admission were enrolled. Three cases with typical findings of Takotsubo cardiomyopathy, such as hypokinesis of the mid-to-apical segment, apical ballooning and hyperkinesis of the basal wall, were excluded. Also, 29 patients were diagnosed with sepsis-induced cardiomyopathy. TTE = transthoracic echocardiography.

The definition of SICM is ejection fraction (EF) <50% and a ≥10% decrease compared to the baseline EF which recovered within 2 weeks, in sepsis or septic shock.^[2]^ If the baseline was unknown, we defined the baseline as increase of >10% compared to the initial EF assessed on admission. The definition of recovery is EF improving to baseline within 2 weeks. Inotropic agents such as dobutamine and epinephrine were not used in any patient before TTE assessment. Cases with typical findings of Takotsubo cardiomyopathy, such as hypokinesis of the mid-to-apical segments, apical ballooning, and hyperkinesis of the basal walls, were excluded as shown in Fig. 1. All clinical data were gathered from the electronic medical records written by residents or attending physicians of the emergency and critical care department. TTE was performed within the first 24 hours of admission by well-trained technicians who had performed at least a 100 TTEs under supervision in the past. The modified Simpson method was used to measure EF.

Our primary outcome was the incidence rate of SICM. Our secondary outcomes were the in-hospital mortality rate and length of ICU stay according to the presence or absence of SICM. Patient characteristics were analyzed with the Wilcoxon signed-rank test for continuous variables, and the χ^2^ test for categorical variables. Logistic regression analysis was performed to estimate odds ratios (ORs) and their 95% confidence intervals (CIs) for the incidence rate of SICM. We *a priori* set age, sex, and lactate levels on admission as the covariates. Analyses were performed using the Stata version 11.2 (Stata, College Station, TX). Two-tailed *P* value of <0.05 was considered to be statistically significant. This study was approved by the Urasoe General Hospital institutional ethics committee.

## 3 Results

In total, 258 patients had sepsis or septic shock. Of these, 210 patients with sepsis or septic shock who underwent echocardiography on admission were enrolled. Of these, 103 patients (49.0%) had sepsis and 107 patients (51.0%) had septic shock. There were no significant differences in sex, age, Acute Physiology and Chronic Health Evaluation (APACHE) II scores, and in-hospital mortality between patients who underwent echocardiography on admission and did not (sex, *P* = 0.93; age, 76 vs 77 years. *P* = 0.34; APACHE II scores: 22 vs 21. *P* = 0.09; in-hospital mortality, *P* = 0.055). Of these, 29 patients (13.8%) were diagnosed with SICM. The study population included 116 males and 94 females, with the average age being 73 years. In total, 22 patients with SICM (76.0%) were male and 7 (24.0%) were female. Patient characteristics are shown in Table 1. The prevalence rate of SICM was significantly higher in male than in female (*P* = 0.02). There was a significant difference in age between patients with SICM and those without SICM (median, 69 vs 77 years. *P* = 0.01). APACHE II scores and SOFA scores were significantly higher in patients with SICM than in those without SICM (median, 27 vs 21. *P* < 0.01; 10 vs 7. *P* < 0.01). Lactate levels on admission were significantly higher in patients with SICM than in those without SICM (median, 4.35 vs 2.90 mmol/L, *P* < 0.01). Peak C-reactive protein (CRP) levels were significantly higher in patients with SICM than in those without SICM (median, 26.3 vs 18.7 mg/dL, *P* < 0.01). There was no significant difference in the in-hospital and 30-day mortality rates between patients with SICM and those without SICM (24.1% vs 12.7%, *P* = 0.15; 20.7% vs 12.1%, *P* = 0.23). The hospitalization and length of ICU stay was significantly longer in patients with SICM than in those without SICM (median, 43 vs 26 and 9 vs 5 days, *P* < 0.01, respectively). The maximum dose of norepinephrine was significantly higher (median, 0.11 vs 0 μg/kg/min, *P* < 0.01) and the use of vasopressin was more common (*P* < 0.01) in patients with SICM than in patients without SICM (Table 2). Among patients with SICM, 10 (34.5%) had respiratory infections, 6 (20.7%) had intra-abdominal infections, 5 (17.2%) had urinary tract infections, 3 (10.3%) had musculoskeletal or soft tissue infections, 1 (3.4%) had a device associated infection, and 1 (3.4%) had a central nervous system infection (Table 3). Blood culture was positive in 18 patients with SICM; 7 had gram-positive cocci (GPC) bacteremia and 9 had gram-negative rod (GNR) bacteremia. There was no significant difference in the rate of bacteremia or the rate of GPC and GNR between patients with SICM and those without SICM (*P* = 0.56 and *P* = 0.53, respectively). The risk for SICM was significantly higher in patients with a history of heart failure (HF) and coronary artery disease (CAD), than in those without a history of HF or CAD (*P* = 0.04 and *P* = 0.01, respectively; Table 1).

**Table 1:**
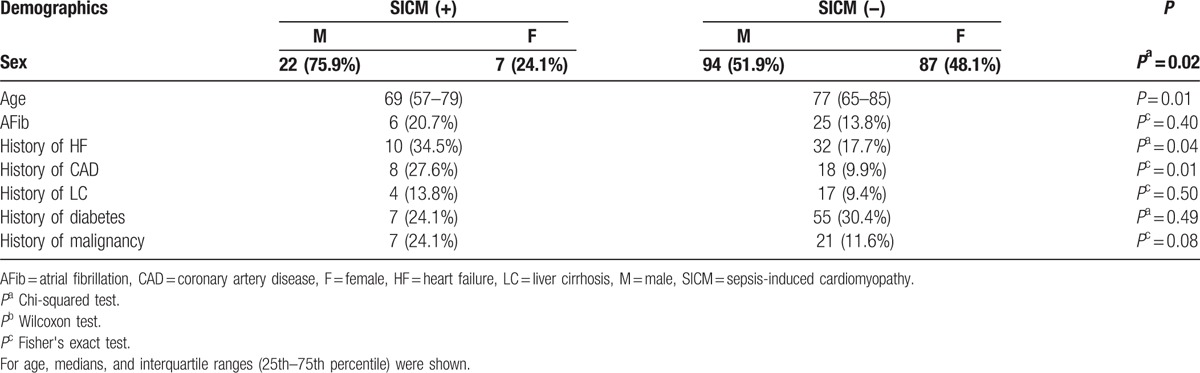
Characteristics of the patients included.

**Table 2:**
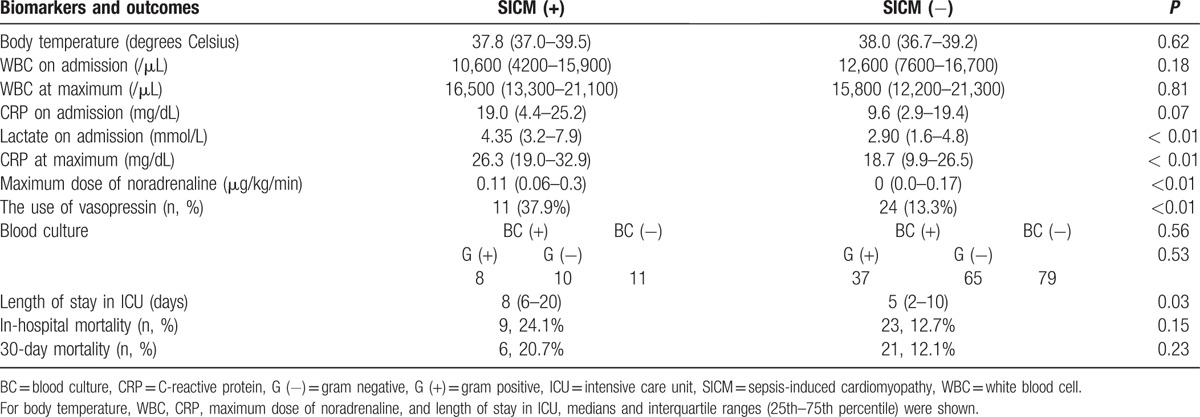
The comparisons of biomarkers and outcomes between the patients with and without sepsis-induced cardiomyopathy.

**Table 3:**
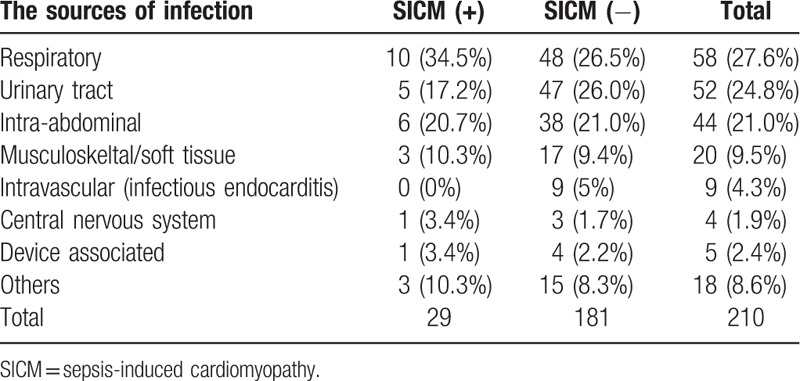
The sources of infection in the participants.

In the multivariate logistic regression analyses, the incidence of SICM was associated with a younger age (OR, 0.96; 95% CI, 0.94–0.99), higher lactate level on admission (OR, 1.19; 95% CI, 1.07–1.33), and history of HF (OR, 3.77; 95% CI, 1.37–10.40), but was not associated with sex (OR, 2.21; 95% CI, 0.85–5.77; Table 4).

**Table 4:**
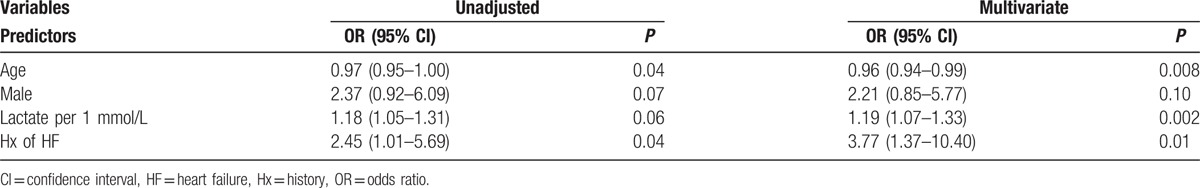
Logistic regression analysis of predictors for sepsis-induced cardiomyopathy.

## 4 Discussion

Previous studies investigated the hemodynamics and molecular pathophysiology of SICM; however, the clinical features remain to be elucidated. To the best of our knowledge, this is the first largest study that revealed the epidemiology of SICM. There were no significant differences in sex, age, APACHE II scores or hospital mortality between patients who underwent TTE on admission or who did not. Thus, the current sample of patients was not susceptible to selection bias. In this study, there were significant differences in the prevalence of SICM with respect to sex predominance, peak CRP level, initial lactate level, APACHE II score, length of hospitalization and ICU stay between patients with SICM and those without SICM. There was a significantly higher risk for SICM in patients with a history of HF and CAD.

The incidence rate of SICM reported in previous studies was 18% to 65%,^[1,7–11]^ while that in this study was 13.8% (29/210). Previous studies suggest that the EF may be affected by the volume status which tends to be higher in hypovolemic patients contributing to the incidence rate varying across other studies. In the present study, we performed early hemodynamic resuscitation termed as early goal-directed therapy (EGDT) for patients with and without SICM.^[12]^

Moreover, the timing of echocardiography may affect the incidence rate of SICM, which may reflect that vasoplegia and left ventricular afterload were corrected in the acute phase.^[13]^ Therefore, we only included patients who underwent TTE within the first 24 hours to minimize the differences of assessment timing. In all patients with decreased EF (<50%), we repeated echocardiography in 2 weeks and checked whether the EF recovered or not.

Some studies suggest that inotropic catecholamine does not improve the outcome of patients with septic shock and may have adverse effects.^[14–18]^ Further, Morelli et al^[19]^ suggested that β-blockade could improve survival by reducing the heart rate without adverse effects. For these reasons, despite the beneficial effects of inotropic catecholamine, it appears that excessive increases in sympathetic tone during sepsis can create adverse effects. Thus, we did not use inotropic agents for patients with SICM.

Peak CRP levels and APACHE II scores were higher in patients with SICM than in those without SICM, suggesting that the inflammation was stronger and more severe in patients with SICM than in those without SICM. Although the mortality rate tended to be higher in patients with SICM than in those without SICM, there were no significant differences in the in-hospital and 30-day mortality between patients with SICM and those without SICM in our study. Considering the inflammation being stronger in patients with SICM than in those without SICM, it could possibly be considered as a paradoxical result. Although the mechanism remains unclear, the SICM-related mortality rate may be low, leading to SICM possibly having a protective effect.^[1]^ The mortality rate was significantly higher in septic patients in a hyperkinetic state than in those in a hypokinetic or normal output state.^[8]^ Conversely, a recent meta-analysis suggested that septic patients with depressed EF were not associated with low mortality rates.^[20]^ However, we must be cautious about the fact that patients were administered inotropic agents such as dobutamine or epinephrine in most studies that were included in the meta-analysis. EF reflects the combination of left ventricular contractility, left ventricular afterload and arguably, the preload.^[21]^ Thus, it may be difficult to clearly describe the association between EF and mortality rates, as well as SICM and mortality rates.

In this study, male, younger age and a history of HF and CAD were considered to be candidate risk factors for SICM. However, high CRP levels, high SOFA scores, high APACHE II scores, and greater use of vasopressors appeared to be a result of SICM rather than risk factors. Decreased coronary flow is not considered to be a main factor in the pathogenesis of SICM,^[2]^ and it is unclear why a history of CAD was more frequent in patients with SICM than in those without SICM. Thus, we did not include the patient's history of CAD in the multivariate logistic regression analysis in this study. Age, sex, lactate levels on admission, and a history of HF were the available data at the admission of septic patients; therefore, we included these 4 variables in the multivariate logistic regression analysis.

The strength of our study is that to the best of our knowledge, this is the first largest study that evaluated the clinical features and epidemiology of SICM. Previous studies have mainly assessed the pathophysiology or hemodynamics of SICM; however, our study focused on the epidemiology and clinical features, including the risk factors, pathogens, and clinical background, which are new and novel findings for SICM.

Our study was not without limitations. First, the number of patients remains small to evaluate the risk factors for SICM. However, we believe that our study presents potential risk factors for SICM, such as history of HF and CAD. Second, this was a single-center study with lower mortality compared to previous clinical studies. It is unclear why the mortality rate was lower than previous reports, while we routinely complied with EGDT as suggested. Martin et al^[22]^ described that in-hospital mortality of patients with sepsis decreased from 27.8% to 17.9% in recent years. Additionally, Kaukonen et al^[23]^ reported that in-hospital mortality of patients with severe sepsis in multiple ICU in Australia and New Zealand decreased 35.0% to 18.4% in recent years. Since our participants were from the recent years between 2013 and 2015, the lower mortality in this study can reflect part of these findings. To confirm our findings or examine the potential risk factors for SICM, a prospective, larger study is warranted.

## 5 Conclusion

SICM developed in 13.8% of patients with sepsis and septic shock. A younger age, higher lactate levels on admission and history of HF were risk factors. The incidence rate of SICM was significantly higher in males and patients with a history of CAD. Furthermore, CRP levels, SOFA scores on admission, and APACHE II scores tended to be higher in patients with SICM than in those without SICM. There was no significant difference in the mortality rates between patients with and without SICM.
